# Emerging novel electronic structure in hydrogen-Arsenene-halogen nanosheets: A computational study

**DOI:** 10.1038/s41598-017-05233-z

**Published:** 2017-07-06

**Authors:** Ming-Yang Liu, Ze-Yu Li, Qing-Yuan Chen, Yang Huang, Chao Cao, Yao He

**Affiliations:** 1 Department of Physics, Yunnan University, Kunming, 650091 China; 2 Department of Physics, Hangzhou Normal University, Hangzhou, 310036 China

## Abstract

Based on first-principles calculations including spin-orbit coupling, we investigated the stability and electronic structure of unexplored double-side decorated arsenenes. It has been found that these new double-side decorated arsenenes, which we call “hydrogen-arsenene-halogen (H-As-X, X is halogen)”, are dynamically stable via the phonon dispersion calculations except H-As-F sheets. In particular, all of H-As-X nanosheets are direct band gap semiconductors with a strong dispersion near the Fermi level, which is substantially different from the previous works of double-side decorated arsenenes with zero band gaps. Our results reveal a new route to change the band gap of arsenene from indirect to direct. Furthermore, we also studied bilayer, trilayer, and multilayer H-As-Cl sheets to explore the effects of the layer number. The results indicate that bilayer, trilayer, and multilayer H-As-Cl sheets display novel electronic structure, namely multi-Dirac cones character, and the Dirac character depends sensitively on the layer number. It is noted that the frontier states near the Fermi level are dominantly controlled by the top and bottom layers in trilayer and multilayer H-As-Cl sheets. Our findings may provide the valuable information about the new double-side decorated arsenene sheets in various practical applications in the future.

## Introduction

In recent years, monolayer group V elements, such as phosphorene and arsenene, have been regarded as new members of two-dimensional (2D) electronic materials family^1–5^. These two intriguing 2D materials have been predicted to be superior to graphene and transition-metal dichalcogenides in optoelectronic applications because of their nonzero band gap and high carrier mobility^2, 6–9^. Similar to phosphorene, theoretical works based on first-principles calculations have proposed that arsenene also has two stable phases in terms of energetics and structural rigidity that is referred to as buckled and puckered arsenene^10^. Both of these two suspended arsenene phases are found to possess indirect band gap and can be tuned to be direct by applying strain^2, 5, 10–13^. Actually, the buckled phase may be more popular than the puckered phase in practical application since the former one is slightly more stable and has a larger band gap (2.49 eV by the hybrid functional levels)^2, 10^. Moreover, for buckled arsenene, it is likely to be manufactured by exfoliating gray arsenic considering the interlayer interaction with the van der Waals (vdW) forces^2, 4, 10^, as in the case of phosphorene. The study on buckled arsenene has become an attractive field. In order to modify the properties of buckled arsenene and give rise to diverse properties, many routes have been adopted^14–20^. Therefore, we choose the buckled arsenene to study in the present work.

It is well known that the 2D materials have a larger surface area so that the surface decoration is a feasible modification to tune the electronic structure, which has been successfully proven in both theory and experiment in graphene and other 2D materials^21–23^. Furthermore, the oxidation behavior of arsenene has also been explored to verify the properties in ambient air^18, 24^. More recently, some studies have revealed that the double-side decorated arsenene, such as AsCH3, AsOH, AsH, and AsX (X is halogen), exhibits Dirac character and unique 2D topological insulator property, and all of them are decorated by the same functional groups on both sides of arsenene in an alternating manner^25–31^. However, to the best of our knowledge, the different functional groups co-decorated arsenene and multilayer decorated arsenene sheets have remained almost unexplored. In the present work, we design a new double-side decorated arsenene that is functionalized by hydrogen on upper/under surface and halogen on under/upper surface, which we call “hydrogen-arsenene-halogen (H-As-X)”, expecting to achieve novel electronic structure for arsenene. Meanwhile, we keep two questions in our mind: Are the new double-side decorated arsenenes also Dirac materials? What will happen when the layer number increases?

In this work, we use first-principles calculations to investigate the stability and electronic structure of these unexplored double-side decorated arsenenes. It has been found that these new double-side decorated arsenenes are dynamically stable via the phonon dispersion calculations except H-As-F sheet. In particular, H-As-X nanosheets are direct band gap semiconductors with a strong dispersion near the Fermi level, which is substantially different from the previous works of double-side decorated arsenenes with zero band gaps. Furthermore, the electronic structures of bilayer, trilayer, and multilayer H-As-Cl sheets are then studied to explore the effects of the layer number. The results indicate that bilayer, trilayer, and multilayer H-As-Cl sheets display novel electronic structures, namely multi-Dirac cones feature, and the Dirac character depends sensitively on the layer number. The frontier states near the Fermi level are dominantly controlled by the top and bottom layers in trilayer and multilayer H-As-Cl sheets. These findings are helpful to obtain the direct-band-gap arsenene and the electronic structure of Dirac character, and provide the valuable information about the properties of arsenene.

## Methods

Our first-principles calculations were based on density functional theory (DFT) as implemented in the Vienna Ab Initio Simulation Package (VASP) codes^32^. For the exchange correlation interaction, we utilized the Perdew-Burke-Ernzerhof (PBE) generalized gradient approximation (GGA) for calculations^33^. The cutoff energy of 500 eV was used for the plane wave basis. We adopted a 21 × 21 × 1 *k*-grid mesh in the Brillouin zone of the primitive unit cell for the geometry optimization, while the *k*-grid mesh was extend up to 41 × 41 × 1 for the self-consistent calculations to obtain more precise electronic structure. As is known to us, the layer structure is affected easily by the vdW forces, and thus we considered the vdW correction PBE-D2 functional^34^ in bilayer, trilayer, and multilayer H-As-X sheets. A vacuum of about 20 Å was introduced to avoid interaction between periodic images of slabs in the z direction. For ionic relaxations, the convergence criterion between two consecutive steps in our self-consistent calculations was 10^−4^ eV, and the criteria for the forces during the ionic relaxation was 0.01 eV Å^−1^. To further discuss the band gap of monolayer H-As-X sheets, the spin-orbit coupling (SOC) was included in the self-consistent calculations of electronic structure. Furthermore, the spin-polarization calculations were also performed to examine the magnetism for H-As-X sheets.

## Results and Discussion

### Optimized atomic structure, energetics, and stability

**Figure 1:**
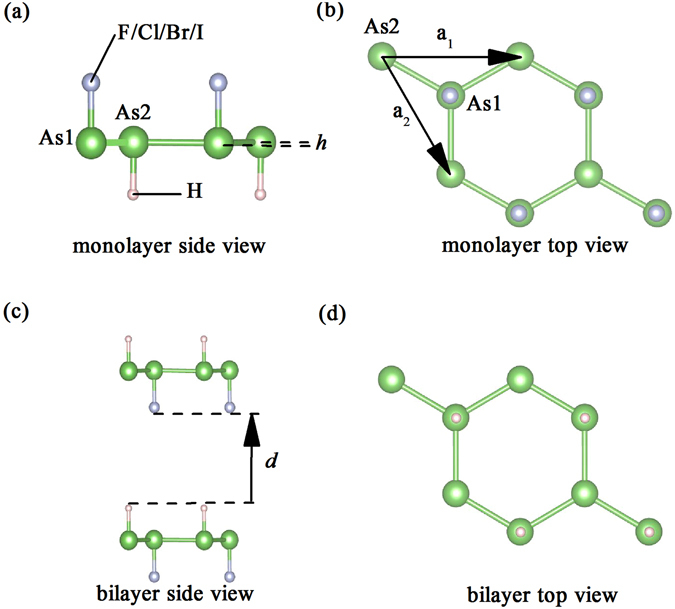
Geometric structures of (**a**) Side view and (**b**) top view of the monolayer H-As-X sheets. (**c**) Side view and (**d**) top view of the bilayer H-As-X sheets via AA-stacking sequence. As, H, and X atoms are represented by ball-and-stick model and distinguished by color, namely green, silver, and pink. The unite cell is marked by ***a***_***1***_ and ***a***_***2***_ vectors.

We first calculated the structural parameters of pristine arsenene, double-side hydrogenated arsenene, and double-side halogenated arsenene to ensure the following investigations, as listed in Table 1, which are in good agreement with previous works^25, 26, 28, 31^. In contrast to the pristine buckled arsenene, H-As-X sheets are nearly planar structure and the symmetry group has changed from D_3_D-3 to C_3_V-1, with the optimized geometry structures shown in Fig. 1. The monolayer H-As-X sheets, as seen in Fig. 1a,b, have two As atoms, one hydrogen atom, and one halogen atom of per unit cell. And all the optimized structural parameters of H-As-X sheets can be seen in Table 2. Because of the surface decoration, we find that the lattice constant, As-As-As bond angle, and As-As bond length, are larger than that of pristine arsenene, while the buckling height is significantly decreased producing a nearly planar structure. The sum of bond angles at each As atom in H-As-X sheets is equal to 359.96°, 359.77°, 359.78°, and 359.56°, respectively. These values are close to the idealized value of 360° for the sp^2^ configuration and much larger than 328° for sp^3^. These important modifications can be understood from the underlying mechanism that the lone-pair electrons of pristine arsenene are bond with hydrogen or halogen when the hydrogen and halogen decorate, which changes from the original hybridization sp^3^ of pristine arsenene to sp^2^, resulting in that the bond angle increases and the buckling height decreases. Compared with double-side hydrogenated and halogenated arsenenes, however, no matter the lattice constant or the As-As bond length, there is only slight change. Furthermore, it is noteworthy that the lattice constant, buckling height, As-As bond length, and As-X bond length, tend to increase monotonously with the X atomic number, while the As-H bond length preserves the same value. This variation results from the fact that, with the increase of X atomic number, the atomic radius increases gradually and the electronegtivity decreases. Similar to H-As-X sheets, the same situation also was obtained in double-side halogenated arsenene except the buckling height as seen in Table 1.

**Table 1:** The optimized structural parameters and band gap of H-As-H and X-As-X: lattice constant *a*, buckling height *h*, sum of bond angle *s θ*, bond lengths *l*_*As-As*_, *l*_*As-H*_ and *l*_*As-X*_, band gap *Eg*.

structure	*a*(Å)	*h*(Å)	*θ* (deg)	*l*_*As-As*_ (Å)	*l*_*As-H*_ (Å)	*l*_*As-X*_ (Å)	*Eg* (meV) with/without SOC
H-As-H	4.64	0.021	359.99	2.68	1.54	/	196/0
F-As-F	4.56	0.117	359.50	2.64	/	1.81	113/0
Cl-As-Cl	4.64	0.055	359.90	2.68	/	2.22	197/0
Br-As-Br	4.64	0.073	359.78	2.68	/	2.38	197/0
I-As-I	4.70	0.150	358.92	2.72	/	2.59	207/0

**Table 2:** The optimized structural parameters and band gap of H-As-X: lattice constant *a*, buckling height *h*, sum of bond angle s *θ*, bond lengths *l*_*As-As*_, *l*_*As-H*_ and *l*_*As-X*_, band gap *Eg*, formation energy *E*_*for*_.

H-As-X	*a*(Å)	*h* (Å)	*θ* (deg)	*l*_*As-As*_ (Å)	*l*_*As-H*_ (Å)	*l*_*As-X*_ (Å)	*Eg* (meV) with/without-SOC	*E*_*for*_ (eV)
H-As-F	4.62	0.033	359.96	2.67	1.55	1.79	25/179	−4.481
H-As-Cl	4.64	0.061	359.77	2.68	1.55	2.20	113/85	−2.814
H-As-Br	4.65	0.076	359.78	2.69	1.55	2.36	95/103	−2.583
H-As-I	4.68	0.102	359.56	2.71	1.55	2.57	95/95	−2.257

**Figure 2:**
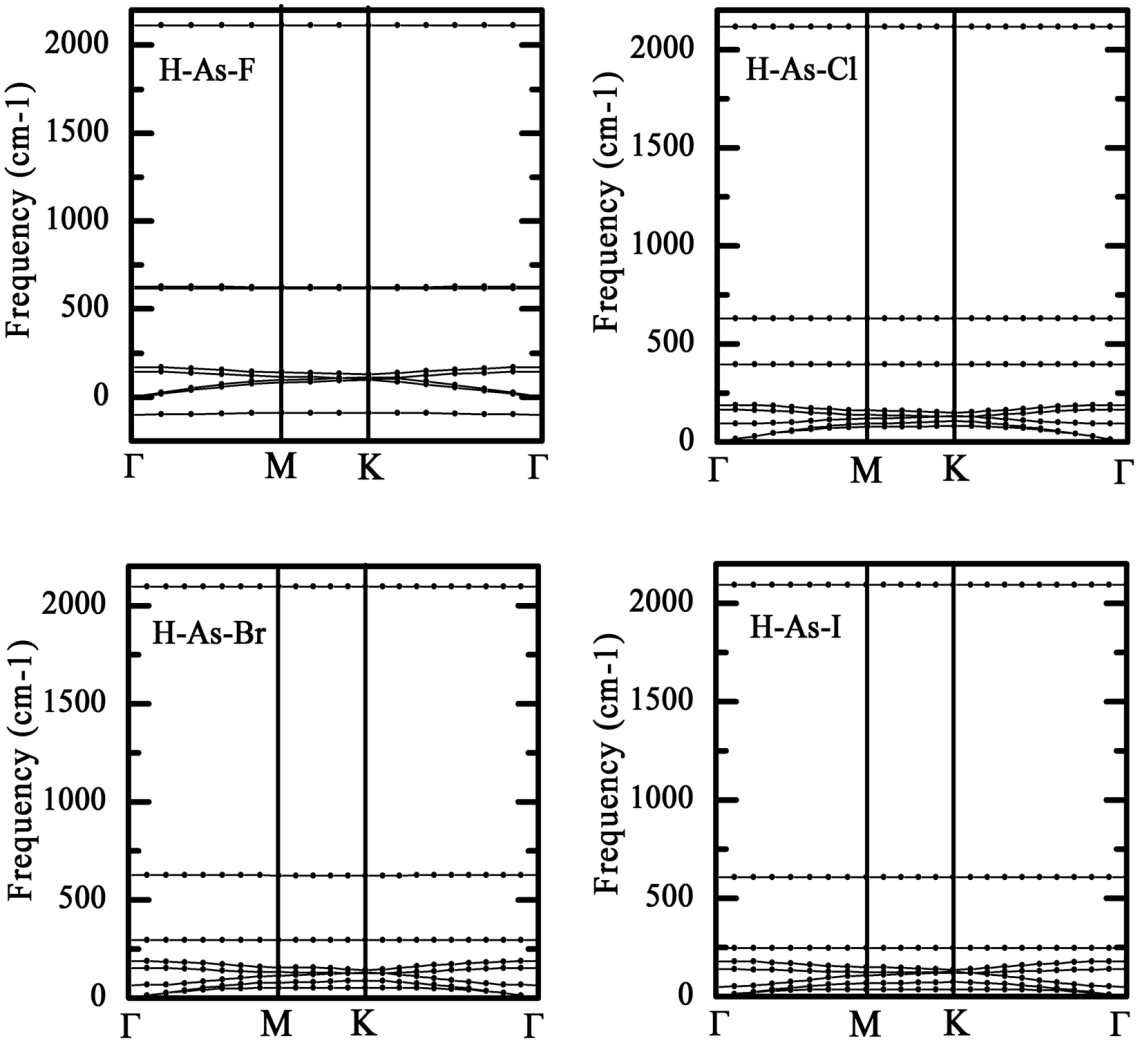
Phonon dispersions of the H-As-X sheets, which are used to examine the kinetic stability.

To examine the energetic feasibility and kinetic stability of H-As-X sheets, the formation energy and phonon dispersion were calculated. The formation energy *E*_*for*_ is defined as *E*_*for*_ = *E*_*H-As-X*_ − (*E*_*As*_ + *E*_*H*_ + *E*_*X*_) (1), where *E*_*H-As-X*_ and *E*_*As*_ are the total energy of H-As-X sheets and pristine arsenene, respectively. *E*_*H*_ and *E*_*X*_ are the per-atom energy of hydrogen and halogen molecules. According to the definition, the larger absolute value of *E*_*for*_ is more exothermic, indicating that it may be more stable and energetically feasible. Unsurprisingly, we notice that the variation of *E*_*for*_ values coincides well with the electronegtivity of halogens, as seen in Table 2. The calculations of kinetic stability are essential to predict the experimental feasibility of H-As-X sheets. The calculated phonon dispersions are plotted in Fig. 2, the absence of soft phonon modes along all momenta demonstrates that H-As-Cl, H-As-Br and H-As-I sheets are kinetically stable. On the contrary, H-As-F sheet may be not dynamically stable owing to the obvious imaginary frequency along Γ-M-K direction even though it has the most negative *E*_*for*_ value.

### Electronic structure of single layer H-As-X sheets

**Figure 3:**
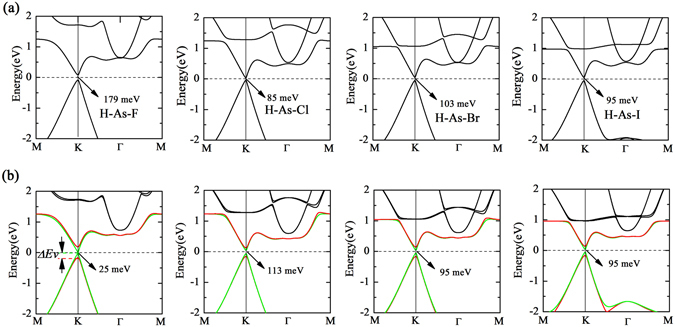
Band structure of the H-As-X sheets (**a**) without SOC and (**b**) with SOC. The Fermi level is set to zero energy and indicated by the dashed line. The degenerated band near the Fermi level is split into two single bands, indicated by red and green lines in (**b**).

**Figure 4:**
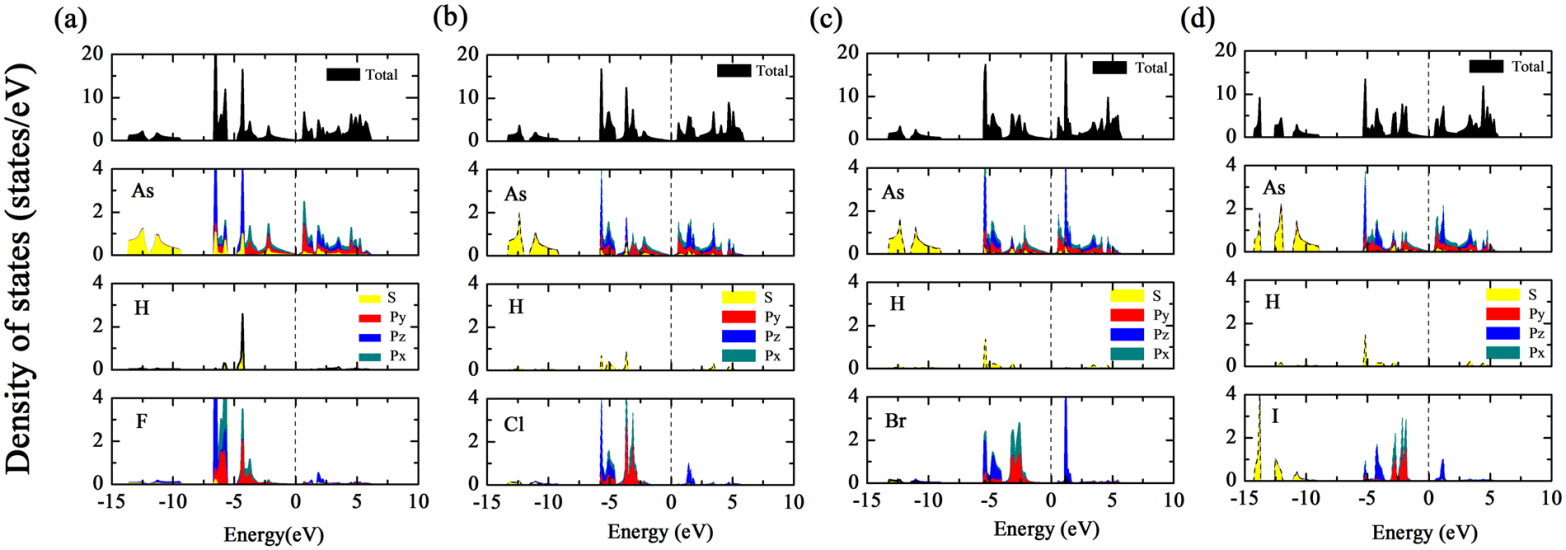
Density of states of (**a**) H-As-F, (**b**) H-As-Cl, (**c**) H-As-Br, and (**d**) H-As-I sheets without SOC. The Fermi level is set to zero energy and indicated by the dashed line.

To find the answers of the question mentioned previously, we then calculated the electronic structure of H-As-X sheets. Interestingly, it is observed that all of the H-As-X sheets have a direct band gap, i.e., 179 meV, 85 meV, 103 meV, 95 meV, which is substantially different from other double-side decorated arsenenes^25, 26, 28, 30^, as shown in Fig. 3a. The direct band gap originates from the fact that the top of valence band shifts from Γ to K and the bottom of conduction band shifts from Γ-M to K. Especially, the states near the Fermi level at K point display a very strong dispersion, indicating exceedingly low effective mass. Since stronger band dispersion is found in H-As-X sheets than in pristine arsenene, we deduce that H-As-X sheets may possess a higher hole mobility than pristine arsenene. In analogy to that the states of other double-side decorated arsenenes near the Fermi level mainly are attributed to p_x_ and p_y_ orbitals of As atoms^25, 27, 30, 31^, the states of H-As-X sheets near the Fermi level are also primarily composed of p_x_ and p_y_ orbitals of As atoms, due to the fact that the p_z_ orbital of As atoms are passivated by H and X atoms (see Fig. 4). But, the p_z_ orbitals of X atoms have an obvious contribution in the lower energy range of conduction band. A similar situation has also been proposed in X-N/Bi-X monolayer by using the tight-binding method in combination with first-principles calculations^27^. From Fig. 4, we find that the pp orbital hybridization between As and X atoms mainly locates in the energy range from −6 eV to −2 eV, and the energy position of sp orbital hybridization between As and H atoms is from −6 eV to −4 eV. Among the H-As-X sheets, H-As-F has the largest direct band gap, as listed in Table 2, but it may be restricted in practical applications as a result of the kinetic instability. It should be mentioned that the relatively narrow band gap of H-As-X sheets is likely underestimated as a common shorting of PBE functional, but still, the electronic structure of the band region is believed to be credible. In addition, we also performed the spin-polarization calculations to examine whether H-As-X sheets possess the magnetic properties. Our results suggest that H-As-X sheets are nonmagnetic, which is consistent with other double-side decorated arsenenes, but different from single-side decorated arsenene that has a small magnetic moment of 0.92 *μ*_*B*_^26^. In what follows, we will reveal the reason why the Dirac character disappears and the direct band gap is formed in H-As-X sheets.

**Figure 5:**
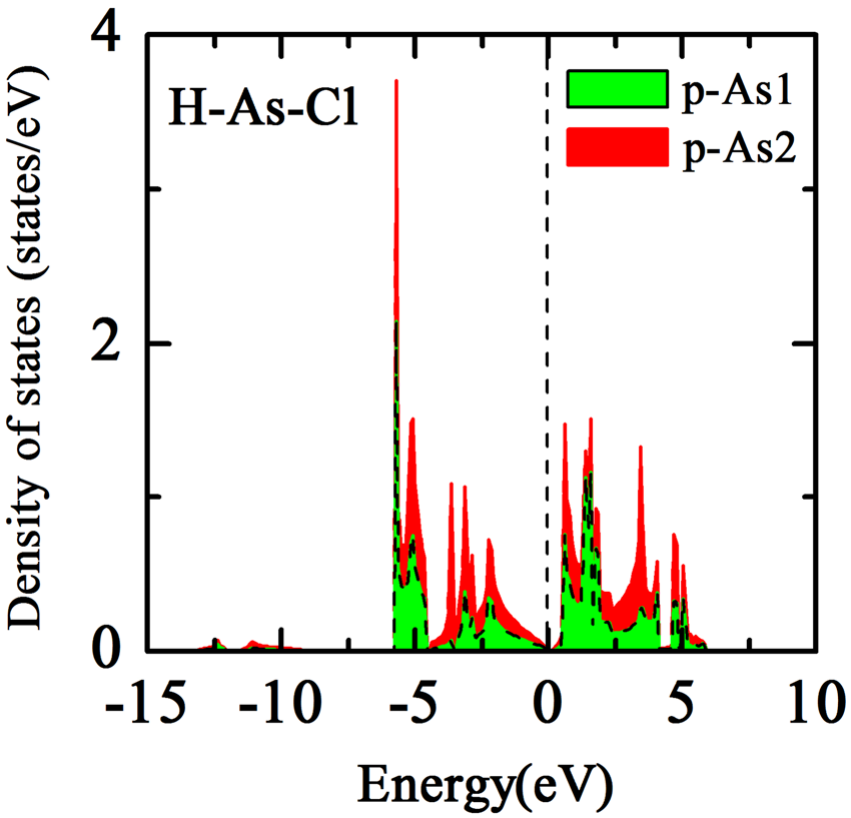
Density of states of the p orbitals of As1 and As2 atoms in H-As-Cl sheets.

As known above, the decorating atoms are on both sides of arsenene in an alternating manner in previously double-side decorated arsenenes. Thus both As1 and As2 atoms (shown in Fig. 1a,b) have the same on-site energy and hybridization between As atom and decorated atom. However, for H-As-X sheets, since the different atoms are separately decorated on both sides of arsenene, the symmetry of sublattice consisting of As1 and As2 is broken. This leads to that the on-site energy is also no longer equal, which can be obviously suggested by the relative movement of the p-energy between As1 and As2 atoms, as shown in Fig. 5. This situation is analogous to the graphene substrated by hexagonal boron nitrogen which breaks the symmetry of carbon sublattice^35–37^. Accordingly, not only the symmetry of sublattice is broken but also As1 and As2 atoms have an entirely different hybridization, which gives rise to the small direct band gap in H-As-X sheets.

Previous works have reported that the double-side decorated arsenene is a quantum spin Hall insulator with sizable energy gap when the SOC is switched on^27, 30, 31^, since the intrinsic SOC of arsenic atom is larger than that of carbon atom and the Dirac character is donated from p_x_ and p_y_ orbitals^29^. Consequently, we further calculated the electronic structure of H-As-X sheets with SOC. It can be obviously found that the degenerated bands near the Fermi level, as depicted in Fig. 3b, are distinctly split into two single bands. The energy difference Δ*Ev* caused by spin splitting at the top of valence band is 158 meV, 87 meV, 108 meV, and 106 meV from H-As-F to H-As-I sheets, respectively. The strongest chemical interaction between F and As atoms gives rise to the largest spin-splitting energy. These spin-splitting induced energy difference which is larger than room energy will have an important influence on carrier transport. These findings may endow H-As-X sheets with great potential in spintronics application. Furthermore, the band gap is decreased or increased for H-As-X sheets with SOC on account of the degenerated band split. For instance, the band gap of H-As-F decreases from 179 meV to 25 meV, while that of H-As-Cl increases from 85 meV to 113 meV. Particularly, the band gap of H-As-I maintains the same value with and without SOC. Although SOC induces the band split, it does not change the strong band dispersion near the Fermi level, exhibiting the robust band dispersion feature in H-As-X sheets.

### Electronic structure of bilayer, trilayer, and multilayer H-As-X sheets

Although the properties of pristine arsenene depend sensitively on the layer number^4^, the double-side decorated arsenene with layers increasing has not been reported. Thus, it is of interest to study the properties of double-side decorated arsenene with layers increasing. Herein, we designed and studied bilayer, trilayer, and multilayer H-As-X sheets, namely layers increasing from two to five, to reveal whether similar character can occur in H-As-X sheets. Two H-As-X sheets are stacked via AA-stacking sequence to form bilayer H-As-X sheets as shown in Fig. 1c,d. Since H-As-Cl sheets are relatively stable and other H-As-X sheets have a similar situation, we mainly focus on bilayer, trilayer, and multilayer H-As-Cl sheets to investigate the effects of the layer number for the sake of brevity. The optimum interlayer distance d of bilayer H-As-Cl sheets is 4.66 Å, which is lower than that of AA-stacking bulk gray arsenic, indicating a stronger interlayer interaction in bilayer H-As-Cl sheets than AA-stacking bulk gray arsenic^4^. The binding energy that is contributed by vdW and chemical interactions between two layers is calculated by comparing the difference of the total energies of the free single H-As-Cl sheets and of the billayer. We defined the binding energy expression as *E*_*b*_ = *2E*_*single*_ − *E*_*bilayer*_ (2). The calculated *E*_*b*_ of bilayer is 0.60 eV, which demonstrates a strong interaction between two layers. Similarly, the *E*_*b*_ of trilayer is obtained to be 0.93 eV, which is larger than that of bilayer owing to the stronger vdW and chemical interactions between layers. We find that the binding energy increases gradually with the layer number, and it is calculated to be a relatively large value 1.59 eV when the layer number is five, corresponding to the strong interlayer interaction. As a consequence, it can be expected that the electronic structure of H-As-Cl would be effectively tuned by the layer number.

**Figure 6:**
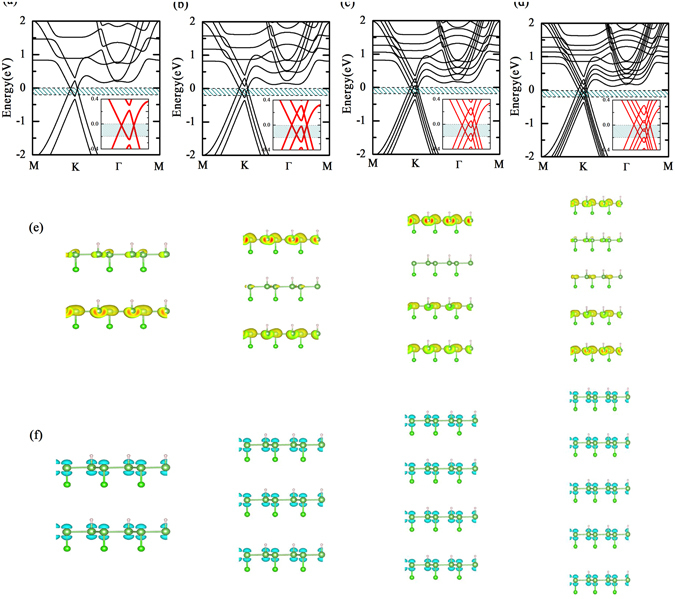
Band structure of (**a**) bilayer, (**b**) trilayer, (**c**) four-layer, and (**d**) five-layer H-As-Cl sheets via AA-stacking sequence. The charge density distributions near the top of valence band in the energy range between the Fermi level and 0.2 eV below it are indicated by the dark cyan shaded region in (**a**) to (**d**), and shown in (**e**). The inserts are the band structure in the energy range from −0.4 eV to 0.4 eV. The charge density difference of VBM of bilayer, trilayer, four-layer, and five-layer H-As-Cl sheets is plot in (**f**).

We present the electronic structure of bilayer, trilayer, and multilayer H-As-Cl sheets in Fig. 6. It can be found surprisingly that bilayer, trilayer, and multilayer H-As-Cl sheets give rise to the novel electronic structure that is not only Dirac character but also possesses multi-Dirac cones. After the passivation of H and Cl atoms, the p_z_ orbital of As atom has been filtered, and thus these Dirac cones originate from the p_x_ and p_y_ orbitals of As atoms, forming in-plane Dirac cone feature^21, 31^. In the case of bilayer H-As-Cl sheets, as presented in Fig. 6a, the strong chemical interactions between layers result in that the lowest conduction band intersects with the highest valence band at both sides of K point, causing two Dirac cones at the Fermi level. In Fig. 6b, as the layer number is three, the overlaps between the conduction band and valence band have large energy range even though it is similar to the bilayer H-As-Cl sheets with two Dirac cones at the Fermi level. We notice that four Dirac cones have been produced at the Fermi level as the layer number increases to four and five, shown in Fig. 6c,d. The mechanism of these exotic Dirac spectrums is that the degeneracy of the valence and conduction bands is lifted because of the interlayer interaction between the H-As-Cl sheets (see Fig. 6), when the single H-As-Cl sheets are brought together to form bilayer, trilayer, or multilayer structures. This leads to the appearance of more bands in the vicinity of the Fermi level. With the H-As-Cl sheet increasing, one extra band appears near the valence band maximum and conduction band minimum such that the number of splitting band is equal to the layer number. Since these splitting bands repel each other, the conduction band intersects with the valence band at both sides of K point, causing Dirac cones near the Fermi level. It is quite important in our finding that the different layer number has similar range for summary of all splitting bands on account of the confinement effect, and thus the number of Dirac cones depends drastically on the layer number. Different from the Dirac character of group IV elements monolayer, the linear dispersion for holes is much longer than that of electrons, for bilayer, trilayer, and multilayer H-As-Cl sheets. This suggests that they are more possible for making p-type field effect devices than for ambipolar ones^21^. Moreover, the p_x_ and p_y_ orbitals of As atoms remain the primary contribution near the Fermi level, which can’t be affected by the layer number. Considering these unexpected multi-Dirac cones features in H-As-Cl sheets which likely prompts the carrier transport efficiency, it should be worth expected that the H-As-Cl sheets as well as other H-As-X sheets apply to the innovative electronic devices.

It is well known that, especially for the H-As-Cl sheets with Dirac character, the properties of frontier states are very important for understanding the carrier transport, which motivates us to further calculate the character of frontier states in bilayer, trilayer, and multilayer H-As-Cl sheets. The frontier states near the top of valence band are calculated in the energy range between the Fermi level and 0.2 eV below it (see Fig. 6a–d). We find that, as depicted in Fig. 6e, the charge density of frontier states mainly derives from As atoms, and no significant contributions are induced by H and Cl atoms. This is consistent with our previous analysis from the orbitals projection. In addition, the frontier states, interestingly, are dominantly controlled by the top and bottom layers in trilayer and multilayer H-As-Cl sheets, whereas the contributions of the intermediate layers are relatively smaller. According to this finding, we can modulate the frontier states by changing the top layers or the bottom layers, to further affect the carrier transport. Finally, we briefly discuss the charge density difference of VBM, which is defined as ${\rm{\Delta }}\rho =\,{\rho }_{H\mbox{-}As\mbox{-}Cl}-\,{\rho }_{decor}-\,{\rho }_{arsenene}$ (3), where ${\rho }_{H\mbox{-}As\mbox{-}Cl}$ is the charge density of H-As-Cl sheets, and *ρ*_*decor*_ and *ρ*_*arsenene*_ are the charge densities of isolated decoration atoms and arsenene at the same position as in the decorated systems, respectively. It is clear from Fig. 6f that all of As atoms deplete charge, indicating the charge transfer from As atom to decoration atoms. This is consistent with their valence charge configuration. Due to the fact that the charge density of VBM mainly comes from As atoms, we do not find an obvious accumulation around decoration atoms.

## Conclusion

In conclusion, our first-principles calculations predict that the new double-side decorated arsenenes, namely H-As-X nanosheets, are dynamically stable except for H-As-F sheets. In contrast to pristine arsenene, all of them form quasi-planar geometry structure with direct band gap. The direct band gaps are obtained to be 179 meV, 85 meV, 103 meV, 95 meV, which is available for the electronic devices at room temperature. We find that the SOC can obviously decrease the band gap of H-As-F sheets. Furthermore, bilayer, trilayer, and multilayer H-As-Cl sheets are also investigated as an example by stacking single H-As-Cl sheets with AA-stacking sequence. The strong chemical interaction between layers gives rise to the Dirac character electronic structure which emerges in bilayer, trilayer, and multilayer H-As-Cl sheets. A significant finding is that the Dirac character will be changed sensitively with the increase of layer number. More importantly, the frontier states near the Fermi level are dominantly controlled by the top and bottom layers in trilayer and multilayer H-As-Cl sheets, which implies that the top and bottom layers may effectively affect the carrier transport. Our findings may provide the valuable information about the new double-side decorated arsenene sheets in various practical applications in the future.
